# m5U-GEPred: prediction of RNA 5-methyluridine sites based on sequence-derived and graph embedding features

**DOI:** 10.3389/fmicb.2023.1277099

**Published:** 2023-10-23

**Authors:** Zhongxing Xu, Xuan Wang, Jia Meng, Lin Zhang, Bowen Song

**Affiliations:** ^1^Department of Public Health, School of Medicine and Holistic Integrative Medicine, Nanjing University of Chinese Medicine, Nanjing, China; ^2^School of AI and Advanced Computing, Xi'an Jiaotong-Liverpool University, Suzhou, China; ^3^Department of Biological Sciences, Xi'an Jiaotong-Liverpool University, Suzhou, China; ^4^Institute of Systems, Molecular and Integrative Biology, University of Liverpool, Liverpool, United Kingdom; ^5^AI University Research Centre, Xi'an Jiaotong-Liverpool University, Suzhou, China; ^6^School of Information and Control Engineering, China University of Mining and Technology, Xuzhou, China

**Keywords:** RNA modification, 5-methyluridine, graph embedding, multi-species, sequence feature

## Abstract

5-Methyluridine (m^5^U) is one of the most common post-transcriptional RNA modifications, which is involved in a variety of important biological processes and disease development. The precise identification of the m^5^U sites allows for a better understanding of the biological processes of RNA and contributes to the discovery of new RNA functional and therapeutic targets. Here, we present m5U-GEPred, a prediction framework, to combine sequence characteristics and graph embedding-based information for m^5^U identification. The graph embedding approach was introduced to extract the global information of training data that complemented the local information represented by conventional sequence features, thereby enhancing the prediction performance of m^5^U identification. m5U-GEPred outperformed the state-of-the-art m^5^U predictors built on two independent species, with an average AUROC of 0.984 and 0.985 tested on human and yeast transcriptomes, respectively. To further validate the performance of our newly proposed framework, the experimentally validated m^5^U sites identified from Oxford Nanopore Technology (ONT) were collected as independent testing data, and in this project, m5U-GEPred achieved reasonable prediction performance with ACC of 91.84%. We hope that m5U-GEPred should make a useful computational alternative for m^5^U identification.

## Introduction

To date, over 170 types of RNA modifications have been identified, occurring on various RNA molecules and influencing nearly every stage of RNA's lifecycle. Scientific research has revealed that these chemical modifications play pivotal roles in numerous critical biological processes (Ontiveros et al., 2019), such as embryonic development (Zhong et al., 2008), cancer development (Zhang et al., 2016a,b), gene-expression regulation (Carlile et al., 2014), and stress response (Wang et al., 2017). Studies have consistently highlighted the significant role of RNA modification in the field of microbiology, encompassing a wide range of aspects, such as the host's m^6^A-marked transcriptome response to the presence of microbiota in mice (Wang et al., 2019), the maintenance of homeostasis between hosts and microbes through modification statuses (Zhuo et al., 2022), and the modulation of host–cell interactions driven by RNA modification (Kostyusheva et al., 2021).

Among over 170 types of chemical markers, RNA 5-methyluridine (m^5^U) is one of the most prevalent and plays a significant role in RNA stability, transcription, and translation. For instance, m^5^U contributes positively to the stability of RNA structures, enhancing their function by modifying base stacking and shaping secondary structures (Agris et al., 2007). Moreover, research studies have demonstrated that m^5^U modification may be associated with virus replication, antiviral immunity, and the development of certain diseases (Väre et al., 2017). Therefore, accurate identification of m^5^U holds profound implications for comprehending fundamental biological processes and functions across different species.

Wet-lab experimental approaches combined with high-throughput sequencing techniques have offered experimentally validated m^5^U sites in multiple species (Xuan et al., 2018; Carter et al., 2019). However, wet-lab approaches can be a costly and time-consuming process; thus, an increasing number of computational efforts have been made, targeting different aspects of biological problems, including phosphorylation prediction (Zhang G. et al., 2023), protein structure prediction (Jumper et al., 2021), drug discovery (Chen et al., 2023), and microbiome studies (Goodswen et al., 2021; Jiang et al., 2022; Yuan et al., 2023). For epitranscriptomic field, a number of bioinformatics databases (Boccaletto et al., 2018; Luo et al., 2021; Song et al., 2021, 2023; Bao et al., 2023; Liang et al., 2023) and *in silico* prediction frameworks (Qiu et al., 2017; Zhai et al., 2018; Chen et al., 2019; Körtel et al., 2021; Xiong et al., 2021; Liang et al., 2022; Song et al., 2022; Yao et al., 2023) have been widely applied. For example, SRAMP was the first sequence-based framework for m^6^A prediction (Zhou et al., 2016), which was also capable of predicting the binding sites of YTHDF1 and YTHDF2. In addition to SRAMP, m6A-Reader (Zhen et al., 2020) was developed specifically to unveil the target specificity and regulatory function of six m^6^A reader proteins (YTHDF1-3, YTHDC1-2, and EIF3A), from which users can identify the putative m^6^A sites involving specific m^6^A enzymes. In terms of m^5^U RNA modification, Jiang et al. (2020) proposed the first sequence-based human m^5^U prediction framework m5UPred, followed by iRNA-m5U targeting yeast transcriptome (Feng and Chen, 2022). The prediction performance of human m^5^U has been further improved by m5U-SVM (Ao et al., 2023) and m5U-autoBio (Yu et al., 2023). In addition, RNADSN was developed by learning the common features between tRNA m^5^U and mRNA m^5^U (Li et al., 2022). These studies together have greatly facilitated the *in silico* identification of m^5^U modification. However, the predictive performance of most computational models is limited by methods that rely on primary sequence-based feature encoding, which does not account for nucleotide frequencies in the training dataset (Hebsgaard et al., 1996), so it is difficult to obtain more complete information from the entire dataset.

**Figure 1:**
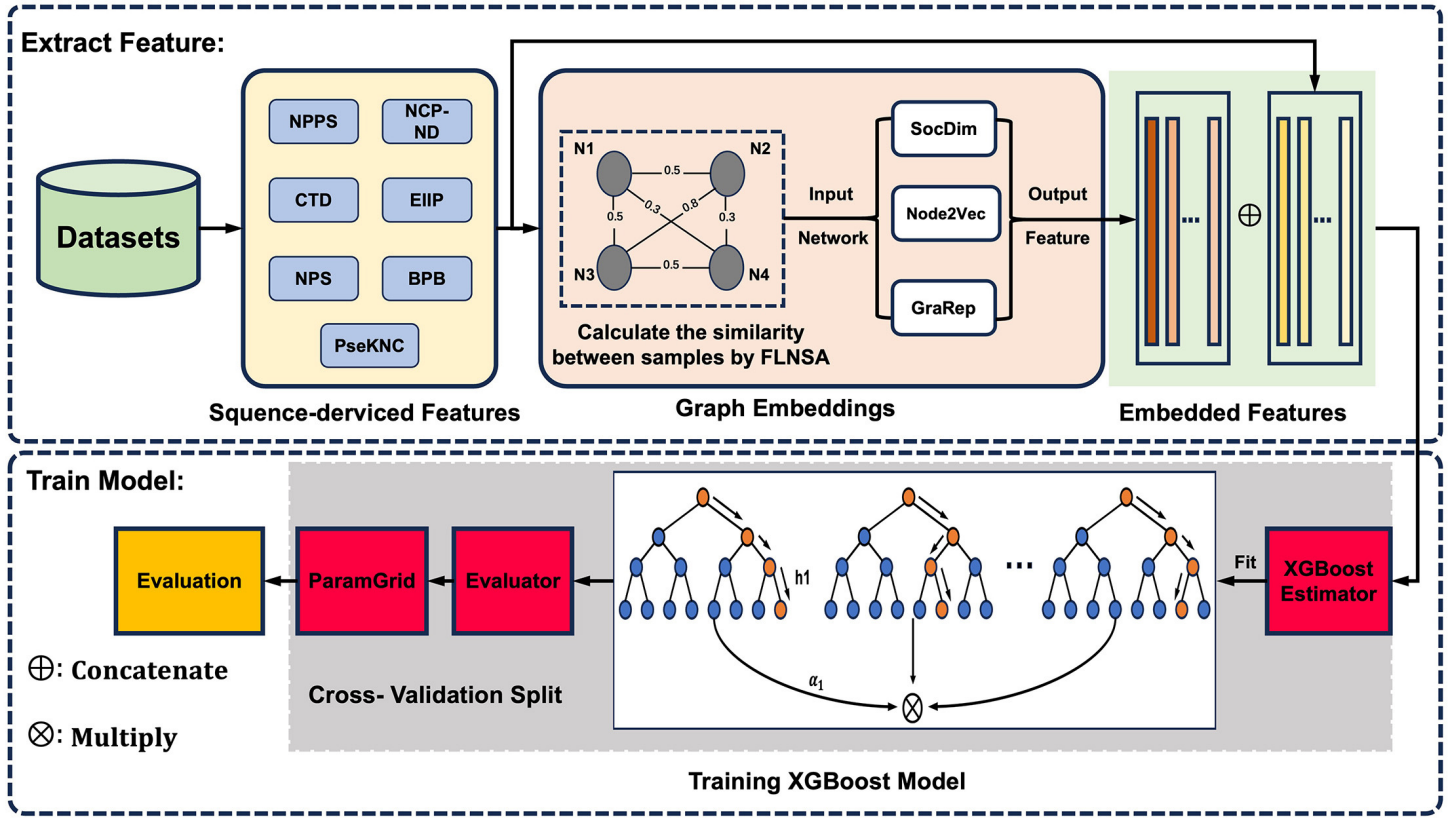
Overall framework of m5U-GEPred. m5U-GEPred was developed by merging sequence-derived information and graph embeddings for accurate m^5^U identification, which used neighborhood-based node embedding through unsupervised learning to extract feature representation containing information from other samples.

To complement sequence-derived features with a more comprehensive understanding of sample information, here, we present m5U-GEPred, the first m^5^U prediction framework that combines sequence-derived features and graph embeddings to identify putative m^5^U modification site. Specifically, m5U-GEPred applies a feature extraction strategy of graph embedding techniques for m^5^U identification, which uses neighborhood-based node embedding technology to obtain feature representations containing information related to other samples through unsupervised learning. With more refined feature extraction, m5U-GEPred outperformed the state-of-the-art m^5^U predictors built on human and yeast transcriptome, with an average AUROC of 0.984 and 0.985, respectively. In addition, we further collected the human m^5^U modification sites deriving from Oxford Nanopore Technology (ONT) as independent testing datasets, and the proposed m5U-GEPred achieved a reasonable prediction performance with ACC of 91.84%. The overall framework of m5U-GEPred is presented in Figure 1.

## Materials and methods

### Benchmark datasets

To build the prediction framework, we obtained the human and yeast m^5^U modification sites from previously published m5UPred (Jiang et al., 2020) and iRNA-m5U (Feng and Chen, 2022), respectively. The experimentally validated human m^5^U sites separated by techniques (miCLIP-seq/FICC-seq) and cell lines (HEK293/HAP1) were extracted for cross-techniques and cross-cell-type validations. Specifically, we used m^5^U sites identified in miCLIP-seq for model development and tested on FICC-seq and vice versa. A total of 3,696 m^5^U sites were obtained from m5UPred to extract the global information of m^5^U data under full transcript mode. In addition to human datasets, the training and testing yeast m^5^U sites were derived from iRNA-m5U, from which 744 positive/negative sites were collected. The positive and negative data were all 41 nt sequences with m^5^Us or unmodified Us in the center.

To further test the performance of our newly proposed framework, the experimentally validated m^5^U sites identified from Oxford Nanopore Technology (ONT) were collected from DirectRMDB (Zhang Y. et al., 2023) and used as independent testing data. Detailed m^5^U datasets used in this study are presented in Supplementary material.

### Model architecture

Inspired by previous studies targeting sequence extraction and graph embedding learning (Zheng et al., 2018; Wang et al., 2021b; Hu et al., 2023), the newly proposed m5U-GEPred can be divided into two main phases (see Figure 1). In phase one, feature extraction involved extracting the sequence-derived information and learning graph embeddings. Seven sequence-based encoding methods were used to convert RNA sequences into numerical vectors. Next, by combining the entire dataset and sequence-derived features, a fast linear neighborhood similarity method constructs a global information network, where samples represent network nodes and edges signify the similarity relationships between the samples. Three unsupervised neighborhood-based node embedding methods, namely, SocDim, Node2Vec, and GraRep, are utilized to learn the characteristics of each node within the global information network, ensuring that graph embedding features of RNA sequences contain relevant information from other samples. Finally, these two types of features are integrated through a feature fusion strategy.

Phase two focused on model building. The data were divided into training and testing datasets, maintaining an 8:2 ratio. The training set was used to train the XGBoost model, while the test set was employed to assess the performance of the predictor. In addition, the cross-technique and cross-cell-type validation were further employed for performance evaluation, where the predictor was trained by m^5^U sites obtained from one technique/cell type and tested on another one. In this project, all the scripts used to build m5U-GEPred are freely accessible, as shown in Supplementary material.

### Sequence-based information

#### Sequence composition and frequency

The nucleotide pair spectrum (NPS) encoding method captures the RNA-seq environment at a specific position by counting the frequency of occurrence of all k-spacer nucleotide pairs in the RNA-seq (Zhou et al., 2016). The *k*-spaced nucleotide pair is *L*_1_{*N*}*L*_2_. Taking sequence “AXXTXG” as an example, “AT” represents a nucleotide pair with a 2 spacing, and “TG” represents a nucleotide pair with a 1 spacing. The window W is the distance from *L*_2_ to *L*_1_. There are N arbitrary nucleotide pairs between *L*_1_ and *L*_2_, so the frequency can be calculated as follows:


$$\begin{array}{l} \text{Frequency}\left(L_{1}\left\{N\right\}L_{2}\right)=\frac{C\left(L_{1}\left\{N\right\}L_{2}\right)}{W-d-1} \end{array}$$

where *C*(*L*_1_{*N*}*L*_2_) is the count of *L*_1_{*N*}*L*_2_ inside a flanking window, and *d* is the space between two nucleotides ranging from 0 to *d*_max_. The encoding method converts a gene sequence into a vector *D*_*NPS*_ with a dimension of 4 × 4 × (*d*_max_+1) = 48. The optimized *d*_max_ was 2 for prediction modes.

The composition, transition, and distribution (CTD) method (Tong and Liu, 2019) is employed to represent global transcribed sequence descriptors. CTD features encompass nucleotide composition, nucleotide transition, and nucleotide distribution, with the latter two serving as RNA secondary structure features essential for classifying coding RNAs. Nucleotide composition (first index C) refers to the percentage composition of each nucleotide present in a transcribed RNA sequence. Nucleotide transitions (second index T) denote the percentage frequency of four nucleotide transitions occurring between adjacent positions. Finally, nucleotide distribution (third index D) illustrates the five relative positions of each nucleotide along the transcribed RNA sequence, specifically at 0% (first), 25%, 50%, 75%, and 100% (last). Both nucleotide transition and nucleotide distribution characteristics play crucial roles in the classification of coding RNAs.

The Bi-profile Bayes feature extraction method describes a Bayesian decision function (Shao et al., 2009). Given an RNA-seq sample *S* = {*s*_1_, *s*_2_, *s*_3_, ⋯ , *s*_*n*_}, S can be divided into two categories, namely, *f*_+_ and *f*_−_, where *f*_+_ and *f*_−_ represent the nucleotide sequence data of known modified sites (positive dataset) and unmodified sites (negative dataset). For each position in the positive and negative datasets, the probability of occurrence of each base (A, C, G, and U) is calculated. According to Bayes' rule, the posterior probability of *S* for these two categories can be given as follows:


$$\begin{array}{l} P(f_{+}|S)\;=\frac{P(S|f_{+})P(f_{+})}{P(S)}\\ P(f_{-}|S)\;=\frac{P(S|f_{-})P(f_{-})}{P(S)} \end{array}$$

Assuming that the prior distribution of category is uniform, namely, *P*(*f*_+_) = *P*(*f*_−_), the formula is as follows:


$$f(S)=sgn\left(\vec{w}•\vec{p}\right)$$

where $\vec{w}=\left({w_{1}}^{+},{w_{2}}^{+},\cdots \;,{w_{n}}^{+},{w_{1}}^{-},\cdots \;,{w_{n}}^{-}\right)$ is weigh vector, and $\vec{p}=\left({p_{1}}^{+},{p_{2}}^{+},\cdots \;,{p_{n}}^{+},{p_{1}}^{-},\cdots \;,{p_{n}}^{-}\right)$ is the posterior probability vector. With respect to training sample, *S, f*(*S*) = 1 corresponds to class *f*_+_ and *f*(*S*) = −1 corresponds to class *f*_−_. In this study, ${p_{1}}^{+},{p_{2}}^{+},\cdots \;,{p_{n}}^{+}$ represents the posterior probability of each nucleotide at each position in *f*_+_ (positive feature space) and ${p_{1}}^{-},\cdots \;,{p_{n}}^{-}$ represents the posterior probability of each nucleotide at each position in *f*_−_ (negative feature space), which we call Bi-profile.

#### Physical and chemical properties

Electron–ion interaction pseudo-potential (EIIP) encoding method (Nair and Sreenadhan, 2006) converts the nucleotides A, G, C, and U in the RNA sequence into their corresponding electron–ion interaction pseudo-potentials by using a simple “EIIP indicator sequence” potential value. Specifically, the EIIP values of nucleotides are as follows: A (adenosine) 0.1260, C (cytosine) 0.1340, G (guanine) 0.0806, and U (uracil) 0.1335. In this method, each nucleotide is assigned a real number associated with its corresponding EIIP value.

#### Local structure information

Pseudo-k-component nucleotide assemblies (PseKNC) are inspired by the PseAAC approach in computational proteomics to represent RNA sequence samples by incorporating global or long-range sequence order effects (Guo et al., 2014).

Converting a gene sequence into vector


$$D={\left[\begin{array}{ccccccc} d_{1} & d_{2} & \cdots & d_{4^{k}} & d_{4^{k}+1} & \cdots & d_{4^{k}+\lambda } \end{array}\right]}^{T}$$

where


$$d_{u}=\{\begin{array}{cc} \frac{f_{u}}{\sum_{i=1}^{u_{k}}f_{i}+w\sum_{j=1}^{\lambda }\theta_{j}} & \left(1\leq u\leq 4^{k}\right)\\ \frac{w\theta_{w-4k}}{\sum_{i=1}^{4k}f_{i}+w\sum_{j=1}^{\lambda }\theta_{j}} & \left(4^{k}\leq u\leq 4^{k}+\lambda \right) \end{array}.$$

In the above equation, $d_{u}\left(u=1,2,\ldots ,4^{k}\right)$is the frequency of k-tuple nucleotide composition (i.e., the combination of k consecutive nucleotides). **w** is the weight factor. λ is the number of RNA sequence-associated cascades. **θ**_**j**_ and **Θ**(**R**_**i**_**R**_**i+1**_**, R**_**i+j**_**R**_**i+j+1**_) are given as follows:


$$\begin{array}{l} \theta_{j}=\frac{1}{L-j-1}\overset{L-j-1}{\underset{i=1}{\sum }}\Theta \left(R_{i}R_{i+1},R_{i+j}R_{i+j+1}\right)\;\left(j=1,2,\ldots ,\lambda ;\;\lambda <L\right)\\ \Theta \left(R_{i}R_{i+1},R_{i+j}R_{i+j+1}\right)=\frac{1}{\mu }\overset{\mu }{\underset{v=1}{\sum }}{\left[P_{v}\left(R_{i}R_{i+1}\right)-P_{v}\left(R_{i+j}R_{i+j+1}\right)\right]}^{2} \end{array}$$

where **μ** is the number of selected local RNA structural features. For a given dinucleotide **R**_**i**_**R**_**i**+**1**_ at position *i*, we assign a numerical value **P**_**v**_(**R**_**i**_**R**_**i**+**1**_) for the v-th local RNA structural property [where (**v** = **1**, **2**, …, **μ**)]. **P**_**ν**_(**R**_**i**+**j**_**R**_**i**+**j**+**1**_) represents the corresponding value for the dinucleotide **R**_**i**+**j**_**R**_**i**+**j**+**1**_ at position *i* + *j*. We consider six local RNA structural properties. The detailed values used for the six physical structural properties were extracted from a previous study (Guo et al., 2014) and are presented in Supplementary Table S1.


$$\text{Translational}\;\text{=}\{\begin{array}{c} \;Rise\;\\ \;Slide\;\\ \;Shift\; \end{array}\;\;\text{Angular}\;\text{=}\{\begin{array}{c} Twist\;\\ \;Roll\;\\ \;Tilt\; \end{array}.\;$$

#### Periodicity features

The nucleotide chemical properties and nucleotide distribution (NCP-ND) feature coding approach combines the chemical properties of nucleotides and their distribution (Bari et al., 2013). Nucleotide distribution is used to measure the density **d**_**j**_ of a specific nucleotide **H**_**j**_ at position **j** and can be derived as follows:


$$\begin{array}{l} d_{j}=\frac{1}{\left|H_{j}\right|}\overset{n}{\underset{j=1}{\sum }}f\left(H_{j}\right) \end{array}$$

where


$$f\left(p\right)=\{\begin{array}{cc} 1 & \;if\;H_{j}=p\in \left\{A,T,C,G\right\}\\ 0 & \text{otherwise} \end{array}$$

**n** is an RNA sequence of length, **j** = **1**, **2**, **3**, …**n** . **D**_**NCP**−**ND**_ is an **n**×**4** dimensional vector.

In the nucleotide chemical property coding scheme, each nucleotide in the RNA sequence exhibits a different function according to its unique chemical structure, thus defining the three coordinate values of the coding scheme.


$$\begin{array}{l} xj=\{\begin{array}{cc} 1 & \;if\;H_{j}\in \left\{A,G\right\}\\ 0 & \;if\;H_{j}\in \left\{C,T\right\} \end{array}\;yj=\{\begin{array}{cc} 1 & if\;H_{j}\in \{A,G\}\\ 0 & \;if\;H_{j}\in \{C,T\} \end{array}\\ \;zj=\{\begin{array}{cc} 1 & \;if\;H_{j}\in \{A,G\}\\ 0 & \;if\;H_{j}\in \{C,T\} \end{array}\; \end{array}$$

#### Nucleotide pair features in sequence

NPPS is a feature representation algorithm based on the position specificity of multi-interval nucleotide pairs (Xing et al., 2017). The frequencies of occurrences of different nucleic acid types are stored at different positions of negative datasets in arrays ${\text{F}}_{\text{s}}^{-}$, ${\text{F}}_{\text{d}}^{-}$:


$$\begin{array}{l} \;F_{s}^{-}=\left[\begin{array}{cccc} t_{s(1,1)}^{-} & t_{s(1,2)}^{-} & \cdots & t_{s(1,C)}^{-}\\ t_{s(2,1)}^{-} & t_{s(2,2)}^{-} & \cdots & t_{s(2,C)}^{-}\\ \vdots & \vdots & \ddots & \vdots \\ t_{s(R,1)}^{-} & t_{s(R,2)}^{-} & \cdots & t_{s(R,C)}^{-} \end{array}\right]\\ F_{d}^{-}=\left[\begin{array}{cccc} T_{d(1,1)}^{+} & T_{d(1,2)}^{+} & \ldots & T_{d(1,C^{2})}^{-}\\ T_{d(2,1)}^{+} & T_{2,2}^{+} & \ldots & T_{d(2,C^{2})}^{-}\\ \vdots & \vdots & \ddots & \vdots \\ T_{d(R^{2},1)}^{+} & T_{16,2}^{+} & \ldots & T_{d(R^{2},C^{2})}^{-} \end{array}\right] \end{array}$$

${\text{F}}_{\text{s}}^{+}$ and ${\text{F}}_{\text{d}}^{+}$ are calculated similarly in the positive dataset. Suppose the *k*-th nucleotide is “U” and the (***k*+ξ**)-th nucleotide is “G”, ${\text{p}}_{\text{k}}^{-}$ can be calculated using the conditional probability formula and the frequency matrix as follows:


$$\begin{array}{l} p_{k}^{-}=\frac{P\left(U\cap G\right)}{P\left(G\right)}=\frac{T_{d\left(UG,k\right)}^{-}}{t_{s\left(G,k+\xi \right)}^{-}} \end{array}$$

The dimension of the vector **D**_**NPPS**_**is** **C**^**2**^**,as** **p**_**k**_**=**${\text{p}}_{\text{k}}^{+}$**-**${\text{p}}_{\text{k}}^{-}$.

#### Graph embedding

To obtain the graph embedding features for each RNA sequence, we build a network encompassing the entire dataset. Within this network, each RNA sequence is considered a node, and the connections between RNA sequences are represented by edges, which typically connect two similar sample nodes. The fast linear neighbor similarity approach (FLNSA) is an efficient method for extracting “sample–sample” similarity (Zhang et al., 2018).

The sequence-derived features, which we extracted above, are converted into n-dimensional feature vectors **x**_**1**_, **x**_**2**_, ⋯ , **x**_**m**_, and each row represents a sample vector and converts the vector into an m^*^n matrix:


$$\begin{array}{l} \underset{W}{\min}\frac{1}{2}∥X-\left(C⊙W\right)X{∥}_{F}^{2}+\frac{\mu }{2}\overset{m}{\underset{i=1}{\sum }}∥\left(C⊙W\right)e{∥}_{F}^{2}\\ \;s.t.\;\left(C⊙W\right)e=e,W\geq 0 \end{array}$$

**C** represents an indicator matrix, where *C*(*i, j*) = 1, if **x**_**j**_ is a neighbor of **x**_**i**_, and **C(i, j) = 0** otherwise, with **C**(**i, i**)** = 0**. The set of neighbors for **x**_**i**_, denoted as **N**(**x**_**i**_), is determined based on the Euclidean distance between **x**_**i**_ and other data points.

Generally, a portion of **x**_**i**_'s neighbors is selected based on distance, and the ratio of neighbor points to all data points is referred to as the neighborhood ratio, denoted as **K**. The Frobenius norm is represented by ∥·∥_*F*_. The column vector **e**, with all elements equal to 1, is denoted as **(1, 1, …, 1)**^**T**^, while ⊙ signifies the Hadamard product. The tradeoff parameter, **μ**, is set to 3. W is an **m × m** weight matrix, where the *i*-th row of W indicates the reconstruction contributions of other data points to the data point**x**_**i**_.

**W**_**ij**_ can be re-calculated as follows:


$$W_{ij}=\{\begin{array}{c} \frac{W_{ij}{\left(XX^{T}+\lambda e^{T}\right)}_{ij}}{{\left((C⊙W)XX^{T}+\mu (C⊙W)ee^{T}\right)}_{ij}}x_{j}\in N\left(x_{i}\right)\\ 0\;x_{j}\notin N\left(x_{i}\right) \end{array}$$

Let **λ=μ*e***.


$$W_{ij}=\{\begin{array}{c} \frac{W_{ij}{\left(XX^{T}+\mu ee^{T}\right)}_{ij}}{{\left((C⊙W)XX^{T}+\mu (C⊙W)ee^{T}\right)}_{ij}}\;x_{j}\in N\left(x_{i}\right)\\ 0\;x_{j}\notin N\left(x_{i}\right) \end{array}$$

Finally, an undirected and unweighted graph is constructed with **w** as the adjacency matrix.

#### SocDim

When multiple relationships are associated with the same network, the SocDim method (Tang and Liu, 2009) can extract the social dimensions of different affiliations of participants hidden in the network and convert them into features for discriminative learning. The method measures the effective amount of community structure in a complex network by measuring the degree of offset (modularity) between interactive platforms and platforms in the network, which involves community detection, a fundamental task in social network analysis.

The modular is defined as follows:


$$\begin{array}{l} Q=\frac{1}{2m}\underset{ij}{\sum }\left[A_{ij}-\frac{d_{i}d_{j}}{2m}\right]\delta \left(s_{i},s_{j}\right) \end{array}$$

Modularity can be reformulated as follows:


$$\begin{array}{l} Q=\frac{1}{2m}Tr\left(S^{T}BS\right) \end{array}$$

When *Q* > 0, it means that soft clustering captures a certain degree of community structure. The modularity matrix is defined as follows:


$$\begin{array}{l} Bx=Ax-\frac{\left(d^{T}x\right)}{2m}d \end{array}$$

where *A* is the interaction matrix, m is the number of nodes, and d is the column vector of nodes. SocDim can extract dimensions (*B*) on the top of the module matrix of the network.

#### Node2Vec

Node2Vec is a graph embedding algorithm designed to learn continuous feature representations of nodes within networks (Grover and Leskovec, 2016). This algorithm aims to learn the mapping of nodes to a low-dimensional feature space, maximizing the preservation of neighborhood information within the network. It employs a biased random walk procedure for efficient exploration of diverse communities, enabling the acquisition of richer representations. Node2Vec formulates the learning of feature vectors in the network as a maximum likelihood optimization problem, which is addressed through the Skip-gram architecture. The objective function is the logarithmic probability of the network neighborhood **N**_*s*_(*u*) by maximizing the observed node *u* as follows:


$$\begin{array}{l} \underset{f}{\max}\underset{u\in V}{\sum }\text{logPr}\left(N_{s}\left(u\right)|f\left(u\right)\right) \end{array}$$

**Figure 2:**
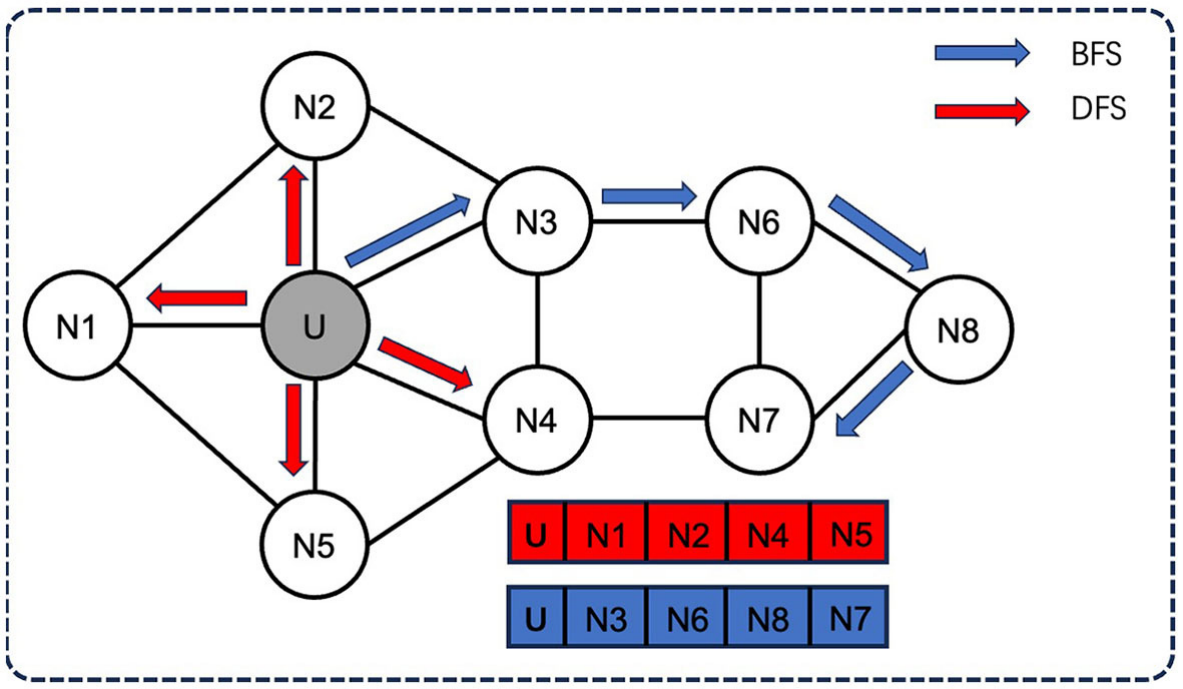
Two sampling strategies of Node2Vec: BFS and DFS. Node2Vec employs two sampling strategies: breadth-first sampling (BFS) and depth-first sampling (DFS), both of which aim to capture different aspects of the network structure. For example, in a neighborhood of size 3, BFS will sample three nodes N1, N2, and N3, while DFS will sample three nodes N4, N5, and N6.

Node2Vec employs two sampling strategies (Figure 2): breadth-first sampling (BFS) and depth-first sampling (DFS), which are based on the network community (nodes directly adjacent to the starting node) and the structural role of the node (the distance from the source node gradually increasing nodes) principle. For example, in a neighborhood of size 3, BFS will sample three nodes N1, N2, and N3, while DFS will sample three nodes N4, N5, and N6.

#### GraRep

GraRep is an algorithm that captures relevant global structural information of a graph by learning the latent representation of vertices on a weighted graph (Cao et al., 2015). The algorithm manipulates different global transformation matrices and extracts various *k*-step relationship information between vertices with different *k* values directly from the graph. First, the *k*-step probability matrix *A*_*k*_ is calculated using the inverse matrix of the degree matrix D and the adjacency matrix S. Then, the *k*-step logarithmic probability matrix *X*_*k*_is calculated and adjusted appropriately, and the positive-logarithmic probability matrix *X*_*k*_ is factorized by SVD to construct the representation vector *W*_*k*_ rows, thereby obtaining the *k*-step representation of each vertex. Finally, all *k*-step representations are concatenated into a global representation.

Furthermore, GraRep designs an accurate on-graph loss function by incorporating non-linear combinations of different local relational information and extending it to support weighted graphs.


$$\begin{array}{l} Y_{i,j}^{k}=W_{i}^{k}\cdot C_{j}^{k}=\log\left(\frac{A_{i,j}^{k}}{\sum_{t}A_{t,j}^{k}}\right)-\log(\beta ) \end{array}$$

### Model construction and performance evaluation

XGBoost is an advanced gradient-boosting algorithm that has consistently displayed outstanding performance (Chen and Guestrin, 2016). Compared with other state-of-the-art gradient boosting techniques, such as CatBoost (Dorogush et al., 2018; Prokhorenkova et al., 2018) and LightGBM (Ke et al., 2017), XGBoost offers the following advantages: (1) It employs a regularized learning framework that prevents overfitting and enhances model generalization; (2) XGBoost utilizes an efficient and parallelized tree construction algorithm to accelerate the training process; (3) it supports the handling of sparse data and missing values, making it suitable for various real-world applications; (4) XGBoost has an extensive range of hyperparameters for tuning, allowing for flexibility and customization to fit specific tasks and datasets. By leveraging these advantages, XGBoost has consistently proven to be a powerful and versatile tool for a wide array of machine learning problems.

For performance evaluation, we applied the following evaluation metrics. In general, the receiver operating characteristic (ROC) curve (sensitivity against 1-specificity) and the area under the ROC curve (AUROC) were used as the primary performance evaluation metrics. In addition, we also calculated sensitivity (Sn), specificity (Sp), Matthews correlation coefficient (MCC), and overall accuracy (ACC) as additional indicators for evaluating the reliability of the model. A five-fold cross-validation was applied on training datasets, while the testing datasets were used for independent testing. Only the m^5^U sites that were not included as part of the training data were selected for independent testing purposes.


$$\begin{array}{lll} S_{n} & = & \frac{TP}{TP+FN}\\ S_{p} & = & \frac{TN}{TN+FN} \end{array}$$

$$\begin{array}{lll} MCC & = & \frac{TP\times TN-FP\times FN}{\sqrt{\left(TP+FP\right)\times \left(TP+FN\right)\times \left(TN+FP\right)\times \left(TN+FN\right)}}\\ ACC & = & \frac{TP+TN}{TP+TN+FP+FN} \end{array}$$

Among them, *TP* represents true positive, while *TN* represents true negative; FP stands for the number of false positive, and *FN* stands for the number of false negative.

## Results

### Performance evaluation in the human transcriptome

The prediction performance of m5U-GEPred in human transcriptome was first evaluated by independent testing datasets and compared with previously published models. For a fair comparison, m5UPred and m5U-autoBio were selected as baseline models based on the same datasets employed. As shown in Table 1, the proposed m5U-GEPred achieved reasonable improvements in prediction performances.

**Table 1:** Prediction performance using independent testing dataset.

**Mode**	**Model**	**Sn (%)**	**Sp (%)**	**ACC (%)**	**MCC**	**AUROC**
Full transcript	m5U-GEPred	93.56	93.90	93.73	0.875	0.984
	m5UPred	87.90	88.80	88.35	0.767	0.956
	m5U-autoBio	93.79	–	92.91	0.858	0.977

### Performance evaluation by cross-technique and cross-cell-type validation

Following m5UPred, we, then, divided the experimentally validated m^5^U sites according to their profiling techniques (miCLIP and FICC-seq) and cell lines (HEK293 and HAP1). For five-fold cross-validation (see Table 2), our newly proposed m5U-GEPred achieved an average AUROC of 0.964 and 0.968 under cross-technique and cross-cell-type validation, respectively, marking reasonable improvements in accuracy compared with the baseline model m5UPred (0.956 and 0.955). In terms of independent testing, m5U-GEPred (0.952 and 0.967) outperformed m5UPred (0.882 and 0.899) with increasing improvements. We also compared the performance of m5U-GEPred with the recently published model m5U-autoBio. When tested on an independent dataset, the performance of m5U-GEPred also outperformed m5U-autoBio (0.883 and 0.921), suggesting the reliability of our newly proposed approach.

**Table 2:** Cross-technique and cross-cell-type validation on full transcript mode.

**Testing method**	**Model**	**Evaluation metric**	**Cross-technique validation**			**Cross-cell-type validation**		
			**miCLIP-Seq**	**FICC-Seq**	**Average**	**HEK293**	**HAP1**	**Average**
Cross validation	m5UPred	Sn	86.70	89.80	88.25	86.26	89.67	87.96
		Sp	86.83	91.37	89.10	87.19	90.48	88.84
		ACC	86.76	90.58	88.67	86.72	80.15	83.44
		MCC	0.735	0.812	0.773	0.735	0.901	0.818
		AUROC	0.946	0.966	0.956	0.942	0.969	0.955
	m5U-GEPred	Sn	67.47	79.93	73.70	72.44	90.22	81.33
		Sp	99.01	98.98	98.99	99.26	91.01	95.14
		ACC	96.14	96.42	96.28	96.79	90.62	93.71
		MCC	0.747	0.840	0.794	0.795	0.812	0.804
		AUROC	0.961	0.967	0.964	0.966	0.970	0.968
Independent dataset	m5UPred	Sn	75.36	56.48	65.92	82.79	57.77	70.28
		Sp	89.23	90.10	89.67	89.62	90.21	89.92
		ACC	82.29	73.29	77.79	86.20	73.99	80.10
		MCC	0.652	0.495	0.574	0.726	0.507	0.617
		AUROC	0.910	0.853	0.882	0.941	0.857	0.899
	m5U-GEPred	Sn	79.49	89.69	84.59	82.79	64.38	73.59
		Sp	93.26	92.38	92.82	92.16	93.14	92.65
		ACC	86.26	90.83	88.55	74.82	78.71	76.77
		MCC	0.735	0.821	0.778	0.752	0.601	0.677
		AUROC	0.944	0.963	0.952	0.944	0.990	0.967

### Independent testing by m^5^U sites generated from nanopore direct RNA sequencing

To further test the performance of our newly proposed framework, the m^5^U sites identified by Oxford Nanopore Technology (ONT) were collected as independent testing data. A total of 98 ONT-derived m^5^U sites were extracted from DirectRMDB, and m5U-GEPred successfully identified 90 of them with an ACC of 91.84%.

### Performance evaluation of m5U-GEPred in yeast transcriptome

In addition to human datasets, the proposed framework was also evaluated by yeast datasets. As shown in Table 3, the performance of m5U-GEPred systemically outperformed iRNA-m5U, which was, to the best of our knowledge, the only available m^5^U predictor in yeast transcriptome. For a fair comparison, the training and testing datasets used to build yeast predictor were exactly the same as iRNA-m5U.

**Table 3:** Prediction performance of yeast m^5^U dataset.

**Source**	**Method**	**Dataset**	**Sn**	**Sp**	**Acc**	**MCC**	**AUROC**
tRNA Transcriptome	iRNA-m5U	tRNA_Dataset	93.88	100	98.82	0.96	0.969
		Dataset 1	93.88	100	96.94	0.94	–
		Dataset 2	93.88	100	96.94	0.94	–
		Dataset 3	93.88	100	96.94	0.94	–
		Dataset 4	93.88	100	96.94	0.94	–
		Dataset 5	93.88	100	96.94	0.94	–
		Dataset 6	91.84	100	95.92	0.92	–
		Dataset 7	95.92	97.96	96.94	0.94	–
		Dataset 8	93.88	97.96	95.92	0.92	–
		Dataset 9	93.88	100	96.94	0.94	–
		Dataset 10	93.88	100	96.94	0.94	–
	m5U-GEPred	tRNA_Dataset	94.00	100	98.82	0.96	0.985
		Dataset 1	95.33	100	97.62	0.95	0.997
		Dataset 2	95.33	100	97.62	0.95	0.993
		Dataset 3	96.00	100	97.96	0.96	0.997
		Dataset 4	96.00	98.67	97.28	0.95	0.997
		Dataset 5	97.33	100	98.64	0.97	0.995
		Dataset 6	94.67	99.33	96.94	0.94	0.992
		Dataset 7	96.67	100	98.30	0.97	0.997
		Dataset 8	94.67	98.00	96.26	0.93	0.997
		Dataset 9	96.00	100	97.96	0.96	0.995
		Dataset 10	96.67	100	98.30	0.97	0.999

In addition, we conducted cross-species validation using human and yeast m^5^U datasets. As shown in Supplementary Table S2, the results indicated that m^5^U modification may exhibit distinct patterns in yeast and human transcriptomes, respectively, suggesting the need to develop species-specific models for m^5^U identification. These findings are consistent with a previous study iRNA-m5U (Feng and Chen, 2022), which only correctly identified 22.45% of human m^5^U sites using yeast training datasets.

### Functional characterization of the predicted m^5^U modification sites using m5U-GEPred

To try to further interpret the prediction results related to biological aspects, we performed a transcriptome-wide prediction of putative m^5^U sites using the newly proposed model. Specifically, we randomly selected 10,000 Us from various types of RNAs of human transcripts and predicted their m^5^U probabilities. Using 0.5 as a cutoff, 224 putative m^5^U modification sites were identified. First, we tried to interpret the prediction results by plotting the overall distribution of the putative m^5^U modification sites using MetaTX (Wang et al., 2021a). The results suggested that putative m^5^U sites were enriched in the 5′UTR (Supplementary Figure S1A). We further performed the gene ontology enrichment analysis of their hosting genes, and as shown in Supplementary Figure S1B, the top 10 biological processes enriched with the predicted m^5^U sites. It may be worth noting that the reason for selecting 0.5 as a general cutoff threshold is that machine learning classifiers usually obtain the lowest empirical rate at a value of 0.5. We further examined the predicted m^5^U sites using different cutoff thresholds (Supplementary Table S3). Additionally, the above results were observed by screening a small portion of the transcriptome (10,000 sites), and these results (~2% of positive results) only suggest a high m^5^U probability at the sequence level (learned from the sequences of positive samples), which should be combined with customized cutoff thresholds (Supplementary Table S3) and wet-lab approaches for final determination. In conclusion, a computational model combined with functional analysis can be a valuable alternative for target identification and result interpretation.

## Conclusion

The accurate identification of 5-methyluridine (m^5^U) modification sites within RNAs holds profound biological significance. In this study, a novel computational approach m5U-GEPred was proposed for m^5^U identification across two independent species. m5U-GEPred combined sequence characteristics and graph embedding-based information, extracting the global information of training data that complemented the local information represented by conventional sequence features. In addition, it may be worth noting that the m^5^U sites detected by experimental approaches may directly relate to reads mapped to expressed genes. This limited the detecting power of modified residues located on low expressed genes under specific conditions. The proposed framework m5U-GEPred accepts sequence information solely as input, which could serve as a useful alternative for m^5^U identification. In addition, the results showed that our newly proposed framework achieved reasonable improvements in prediction performance, compared with the state-of-the-art models developed in human and yeast transcriptome, respectively.

Nevertheless, the proposed m5U-GEPred was developed by combining sequence-based features and graph embedding information, which achieved enhanced prediction performance. The enhanced results suggest that the experimentally identified m^5^U modification sites may have a strong sequence pattern, but the reverse may not necessarily be true (the sequence may be just one of the key features for determining m^5^U). Consequently, machine learning models provide suggestion for the potentially modified residues based on their learned features, which would significantly narrow down the range of target interests (but still a wider range than final experimental identification) for further wet-lab experiments. Consequently, the m^5^U prediction framework and its applications can be further expanded by incorporating the latest sequencing data and binding regions of m^5^U-related enzymes to enable accurate m^5^U identification under different biological contexts, such as target-specific m^5^U prediction.

## Data availability statement

The original contributions presented in the study are included in the article/Supplementary material, further inquiries can be directed to the corresponding author.

## Author contributions

ZX: Methodology, Writing–original draft. XW: Software, Writing–review and editing. JM: Conceptualization, Writing–review and editing. LZ: Conceptualization, Writing–review and editing. BS: Conceptualization, Data curation, Funding acquisition, Supervision, Writing–review and editing.
