# Supplementary material for: m5U-GEPred: prediction of RNA 5-methyluridine sites based on sequence-derived and graph embedding features
Source: Front Microbiol. 2023 Oct 23;14:1277099. doi: 10.3389/fmicb.2023.1277099 (PMC10627201; doi:10.3389/fmicb.2023.1277099)
Supplement: Supplementary file 1 [file Data_Sheet_1.ZIP › Supplementary Files/Supplementary Materials.docx]

**Supplementary Materials**

**Table S1 Values used for the six physical structural properties**

| Dinucleotide | Rise  P_1_(R_i_R_i+1_) | Slide  P_2_(R_i_R_i+1_) | Shift  P_3_(R_i_R_i+1_) | Twist  P_4_(R_i_R_i+1_) | Roll  P_5_(R_i_R_i+1_) | Tilt  P_6_(R_i_R_i+1_) |
| --- | --- | --- | --- | --- | --- | --- |
| AA | 7.65 | 2.26 | 1.69 | 0.026 | 0.020 | 0.038 |
| AC | 8.93 | 3.03 | 1.32 | 0.036 | 0.023 | 0.038 |
| AG | 7.08 | 2.03 | 1.46 | 0.031 | 0.019 | 0.037 |
| AT | 9.07 | 3.83 | 1.03 | 0.033 | 0.022 | 0.036 |
| CA | 6.38 | 1.78 | 1.07 | 0.016 | 0.017 | 0.025 |
| CC | 8.04 | 1.65 | 1.43 | 0.026 | 0.019 | 0.042 |
| CG | 6.23 | 2.00 | 1.06 | 0.014 | 0.016 | 0.026 |
| CT | 7.08 | 2.03 | 1.46 | 0.031 | 0.019 | 0.037 |
| GA | 8.56 | 1.93 | 1.32 | 0.025 | 0.020 | 0.038 |
| GC | 9.53 | 2.61 | 1.20 | 0.025 | 0.026 | 0.036 |
| GG | 8.04 | 1.65 | 1.43 | 0.026 | 0.019 | 0.042 |
| GT | 8.93 | 3.03 | 1.32 | 0.036 | 0.023 | 0.038 |
| TA | 6.23 | 1.20 | 0.72 | 0.017 | 0.016 | 0.018 |
| TC | 8.56 | 1.93 | 1.32 | 0.025 | 0.020 | 0.038 |
| TG | 6.38 | 1.78 | 1.07 | 0.016 | 0.017 | 0.025 |
| TT | 7.65 | 2.26 | 1.69 | 0.026 | 0.020 | 0.038 |

**Table S2. Cross-species validation**

| Training species | Testing species | Accuracy (%) |
| --- | --- | --- |
| Human | Yeast | 50.98 |
| Yeast | Human | 50.05 |

**Table S3. Putative m^5^U sites identified using different cut-off thresholds**

| Cut-off  thresholds | No. predicted  m^5^U positive sites | Identified  frequency (%) |
| --- | --- | --- |
| 0.5 | 224 | 2.24 |
| 0.6 | 140 | 1.40 |
| 0.7 | 81 | 0.81 |
| 0.8 | 43 | 0.43 |

**Figure S1. Characterization of the predicted m^5^U modification sites.** A) Distribution of the putative m^5^U sites predicted by m5U-GEPred. B) Biological processes associated with the predicted m^5^U sites. It may be worth noting that, the above results were observed by randomly selected 10,000 Us from human transcripts and predicted their m^5^U probabilities using m5U-GEPred.
